# Behavioral evidence for the differential regulation of p-p38 MAPK and p-NF-κB in rats with trigeminal neuropathic pain

**DOI:** 10.1186/1744-8069-7-57

**Published:** 2011-08-05

**Authors:** Min K Lee, Seung R Han, Min K Park, Min J Kim, Yong C Bae, Sung K Kim, Jae S Park, Dong K Ahn

**Affiliations:** 1 Department of Oral Physiology, School of Dentistry, Kyungpook National University, Daegu, Korea; 2 Department of Oral Anatomy, School of Dentistry, Kyungpook National University, Daegu, Korea; 3 Department of Endodontics, School of Dentistry, Kyungpook National University, Daegu, Korea; 4 Department of Physiology, School of Medicine, Kyungpook National University, Daegu, Korea

## Abstract

**Background:**

We investigated the differential regulation of p-p38 MAPK or p-NF-κB in male Sprague-Dawley rats with inferior alveolar nerve injury resulting from mal-positioned dental implants. For this purpose, we characterized the temporal expression of p-p38 MAPK or p-NF-κB in the medullary dorsal horn and examined changes in nociceptive behavior after a blockade of p-p38 MAPK or p-NF-κB pathways in rats with trigeminal neuropathic pain.

**Results:**

Under anesthesia, the left lower second molar was extracted and replaced with a mini dental implant to intentionally injure the inferior alveolar nerve. Western and immunofluorescence analysis revealed that p-p38 MAPK is upregulated in microglia following nerve injury and that this expression peaked on postoperative day (POD) 3 through 7. However, the activation of p-NF-κB in astrocyte peaked on POD 7 through 21. The intracisternal administration of SB203580 (1 or 10 μg), a p38 MAPK inhibitor, on POD 3 but not on POD 21 markedly inhibits mechanical allodynia and the p-p38 MAPK expression. However, the intracisternal administration of SN50 (0.2 or 2 ng), an NF-κB inhibitor, on POD 21 but not on POD 3 attenuates mechanical allodynia and p-NF-κB expression. Dexamethasone (25 mg/kg) decreases not only the activation of p38 MAPK but also that of NF-κB on POD 7.

**Conclusions:**

These results suggest that early expression of p-p38 MAPK in the microglia and late induction of p-NF-κB in astrocyte play an important role in trigeminal neuropathic pain and that a blockade of p-p38 MAPK at an early stage and p-NF-κB at a late stage might be a potential therapeutic strategy for treatment of trigeminal neuropathic pain.

## Background

Injuries of the peripheral nerve often result in neuropathic pain, which is characterized by allodynia, hyperalgesia or spontaneous pain. These injuries may affect the activity of spinal glial cells, which are involved in the pathogenesis of neuropathic pain [1]. The spinal glial cells, mainly comprising microglia and astrocyte, are also the most abundant immune cells in the central nervous system. Following peripheral nerve damage, resting microglia and astrocyte are converted to an activated state through a series of cellular and molecular changes [2,3]. In addition, activated microglia and astrocyte participate in the release of pro-inflammatory cytokines such as interleukin-1 beta (IL-1β), interleukin-6 (IL-6), or tumor necrosis factor-alpha (TNF-α), which may augment nociceptive signaling in the spinal cord [4].

Recently, p38 mitogen-activated protein kinase (p38 MAPK) was found to contribute to neuropathic pain in several animal models. Intrathecal injections of p38 MAPK inhibitors were shown to reverse mechanical allodynia and thermal hyperalgesia in rats with an L5 spinal nerve ligation [5]. In addition, the activation of microglial p38 MAPK following an L5 spinal nerve transaction is reduced by minocycline, a microglia inhibitor, or SB203580, a p38 MAPK inhibitor [6]. Emerging evidence also now indicates that the activation of nuclear factor kappa B (NF-κB) following nerve injury is related to the generation of neuropathic pain. Spinal nerve ligation increases the expression of phospho-NF-κB (p-NF-κB) in astrocyte and this activated NF-κB participates in tactile allodynia [7]. Intrathecal pretreatment with NF-κB inhibitors attenuates the allodynia produced by sciatic inflammatory neuropathy [8] and L5 ventral root transaction [9]. However, although the accumulating evidence from diverse animal models indicates that the activation of p38 MAPK and NF-κB plays an important role in neuropathic pain, it remains unknown whether these molecules contribute to the development or modulation of behavioral responses in trigeminal neuropathic pain.

Recently, Han et al. reported that inferior alveolar nerve injury induced by the mal-positioning of dental implants produces prolonged mechanical allodynia in the trigeminal territory in rats [10]. In our present study, we investigated the differential regulation of phospho-p38 (p-p38) MAPK and p-NF-κB in this same rat model. We examined changes in temporal expression of p-p38 MAPK and p-NF-κB in the medullary dorsal horn and also evaluated nociceptive behavior in the subject animals following a blockade of p38 MAPK and NF-κB activation. In addition, we investigated whether the p38 MAPK or NF-κB pathways participate in the antinociceptive action of dexamethasone.

## Results

### Differential expression of p-p38 MAPK and p-NF-κB

**Figure 1:**
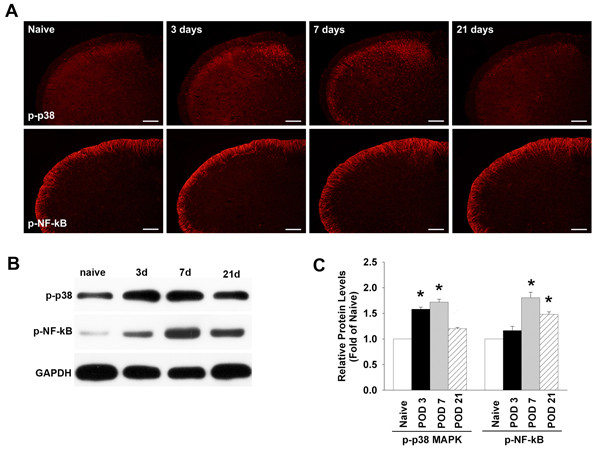
**Changes in temporal expression of p-p38 MAPK and p-NF-κB**. The expression of p-p38 MAPK and p-NF-κB is increased in the rat medullary dorsal horn after inferior alveolar nerve injury induced by mal-positioned dental implants (A). The levels of p-p38 MAPK peaked on POD 3 and 7 and those of p-NF-κB peaked on POD 7 and 21, compare to the naïve group. Western blotting analysis showed the early activation of p38 MAPK and late activation of NF-κB (B). Quantitative analysis of western blotting results revealed that p-p38 MAPK expression increases significantly on POD 3 and 7 and that p-NF-κB expression is markedly upregulated on POD 7 and 21 compared to the naïve group (C). *P < 0.05, naive- *vs. *nerve injured- group. Scale bars, 200 μm

Figure 1 illustrates changes in temporal expression of p-p38 MAPK and p-NF-κB in the medullary dorsal horn in rats after the inferior alveolar nerve injury produced by the placement of mal-positioned dental implants. The sham-treated rats did not show any changes in the expression of these factors as compared to the naïve group (data not shown). However, the expression of p-p38 MAPK significantly increased following nerve injury on postoperative day (POD) 3 and was maintained at this level by POD 7 when compared to the naïve group. Unlike p-p38 MAPK, however, the p-NF-κB peaked on POD 7 and persisted on POD 21 (Figure 1A). Western blotting analysis confirmed that the increases in the phosphorylation of p38 MAPK and NF-κB are time-dependent following nerve injury. Significant increases in the expression of p-p38 MAPK on POD 3 through POD 7 and p-NF-κB on POD 7 through POD 21 were detected by immunoblotting when compared to the naïve group (P < 0.05; Figure 1B, 1C).

### Effects of treatment with SB203580

**Figure 2:**
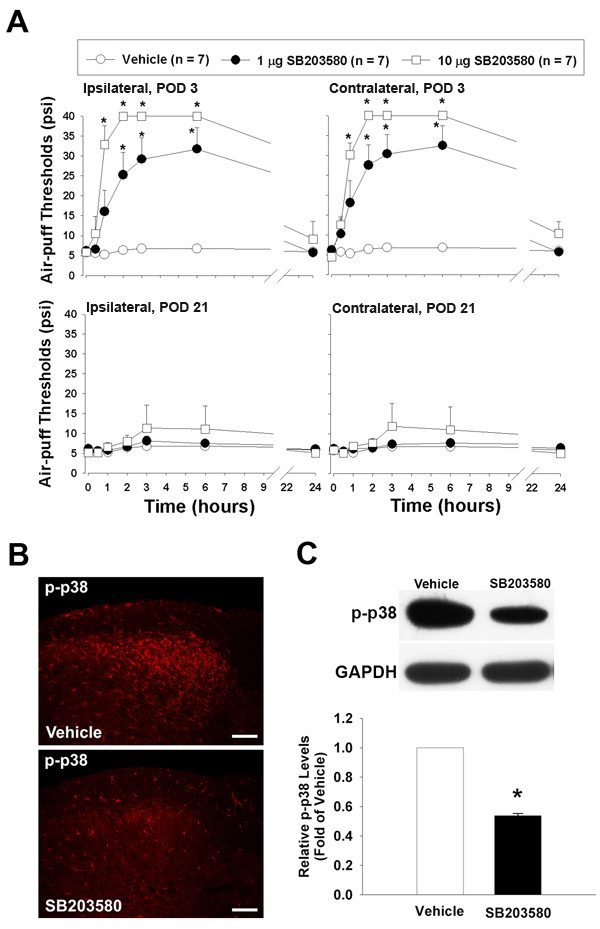
**Antinociceptive effects of SB203580, a p38 MAPK inhibitor**. Effects of SB203580 on mechanical allodynia in rats with trigeminal neuropathic pain produced by mal-positioned dental implants (A). The intracisternal administration of SB203580 (1 or 10 μg) inhibited mechanical allodynia on the ipsilateral (left) and contralateral side (right) on POD 3 in a dose-dependent manner. These anti-allodynic responses persisted for six hours and were recovered within 24 hours. On POD 21 however, SB203580 did not produce anti-nociceptive effects (lower panel). The intracisternal administration of SB203580 (10 μg) attenuated p38 MAPK activation on POD 3 following inferior alveolar nerve injury as compared to the vehicle group (B). Western blotting analysis showed decreases in p-p38 MAPK expression following the injection of SB203580 as compared to the vehicle treatment (C). *P < 0.001, vehicle- vs. SB203580-treated group. Scale bars, 50 μm.

The effects of SB203580 treatments on mechanical allodynia in rats with trigeminal neuropathic pain induced by inferior alveolar nerve injury are illustrated in Figure 2. The intracisternal administration of SB203580 (1 or 10 μg), a p38 MAPK inhibitor, inhibited mechanical allodynia in a dose-dependent manner when it was administered on POD 3 (F_(2, 18) _= 34.056, P < 0.001). These anti-allodynic responses persisted for six hours and recovered within 24 hours. Mirror image mechanical allodynia was also attenuated by the intracisternal administration of SB203580. However, intracisternally injected SB203580 did not alter the air-puff threshold when it was administered on POD 21 (Figure 2A). On POD 3, the intracisternal administration of SB203580 (10 μg) significantly reduced p-p38 MAPK expression in the ipsilateral medullary dorsal horn as compared to the vehicle group (Figure 2B). Western blotting analysis also revealed the inhibition of p38 MAPK phosphorylation after treatment with SB203580 (Figure 2C).

### Effects of SN50

**Figure 3:**
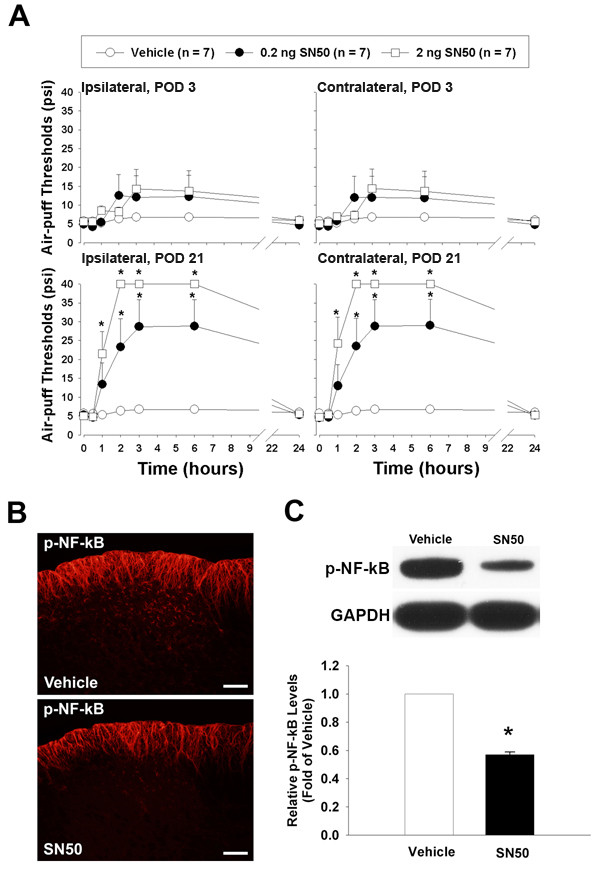
**Antinociceptive effects of SN50, an NF-κB inhibitor**. Effects of SN50 on mechanical allodynia produced by mal-positioned dental implants in rats (A). On POD 3, SN50 did not produce anti-nociceptive effects (upper panel). The intracisternal administration of SN50 (0.2 or 2 ng) inhibits mechanical allodynia on the ipsilateral (left) and contralateral side (right) on POD 21 in a dose-dependent manner. These anti-allodynic responses persist for six hours and are recovered within 24 hours. The intracisternal administration of SN50 (2 ng) attenuates NF-κB activation on POD 21 following inferior alveolar nerve injury as compared to the vehicle group (B). Western blotting analysis showed decreases in p-NF-κB expression following injection of SN50 as compared to the vehicle treatment (C). *P < 0.001, vehicle- vs. SN50-treated group. Scale bars, 50 μm.

The effects of treatment with SN50 on mechanical allodynia in our rat model are shown in Figure 3. The intracisternal administration of SN50 (0.2 or 2 ng) on POD 21 but not on POD 3 produced significant anti-allodynic effects compared to the vehicle group (F_(2, 18) _= 21.185, P < 0.001). These anti-allodynic responses persisted for six hours and recovered within 24 hours. Mirror image mechanical allodynia was also attenuated by SN50 in this experiment (Figure 3A). On POD 21, the intracisternal administration of SN50 (2 ng) significantly reduced p-NF-kB expression in the ipsilateral medullary dorsal horn as compared to the vehicle group (Figure 3B). Western blotting analysis confirmed the inhibition of NF-kB phosphorylation after treatment with SN50 (Figure 3C).

### Co-localization of p-p38 MAPK and p-NF-kB

**Figure 4:**
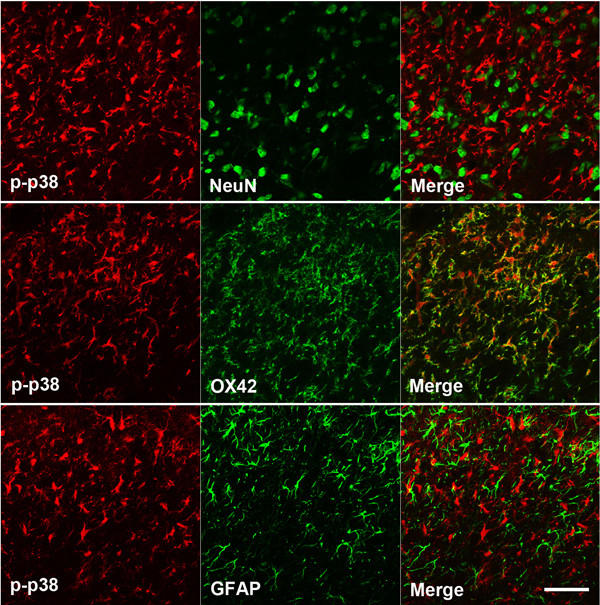
**Double immunofluorescent staining of p-p38 MAPK**. P-p38 MAPK (red) immunoreactive cells were found to mainly co-localize with OX42 (green), but not NeuN (green) or GFAP (green) in the rat medullary dorsal horn following inferior alveolar nerve injury. Scale bars, 50 μm.

**Figure 5:**
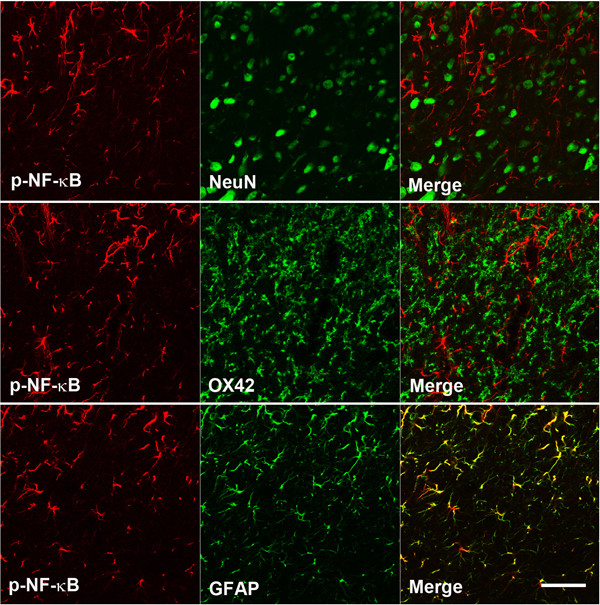
**Double immunofluorescent staining of p-NF-kB**. All p-NF-kB (red) immunoreactive cells co-localize with GFAP (green), but not NeuN (green) or OX42 (green) in the rat medullary dorsal horn following inferior alveolar nerve injury. Scale bars, 50 μm.

We performed double immunostaining for p-p38 MAPK on POD 3 and p-NF-κB on POD 21 (Figure 4, 5). The results revealed that p-p38 MAPK co-localizes with OX42, a marker of microglia, but not with neuronal nuclei (NeuN), a neuronal marker or glial fibrillary acidic protein (GFAP), an astrocyte marker, in the medullary dorsal horn (Figure 4). In contrast, most of the p-NF-κB immunoreactive cells were found to co-localize with GFAP in the medullary dorsal horn (Figure 5).

### Effects of dexamethasone on p-p38 MAPK and p-NF-κB expression

**Figure 6:**
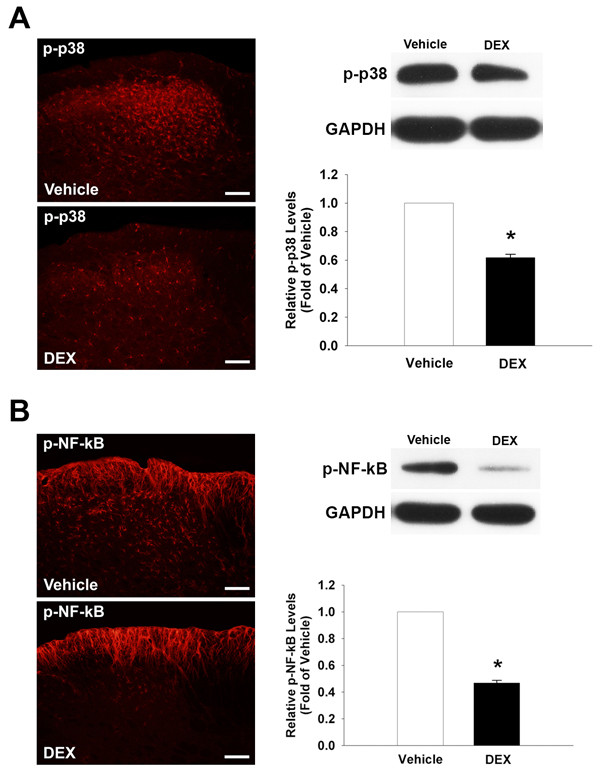
**Effects of dexamethasone on the expression of p-p38 MAPK and p-NF-κB**. The intraperitoneal administration of dexamethasone (25 mg/kg) attenuates p-p38 MAPK expression on POD 7 following inferior alveolar nerve injury as compared to the vehicle group (A). Western blotting analysis also revealed a significant decrease in p-p38 MAPK expression as compared to the vehicle treatment. The intraperitoneal administration of dexamethasone (25 mg/kg) also attenuates p-NF-κB expression on POD 7 following inferior alveolar nerve injury as compare to the vehicle group (B). Western blotting analysis also confirmed this significant decrease in p-NF-κB expression. *P < 0.05, vehicle- vs. drug-treated group. Scale bars, 50 μm.

We recently demonstrated that the early administration of dexamethasone significantly blocks mechanical allodynia and hyperalgesia produced by mal-positioned dental implants [10]. To evaluate in our current study whether the antinociception produced by dexamethasone is associated with p38 MAPK or NF-κB phosphorylation, we performed immunostaining following the intraperitoneal administration of 25 mg/kg of dexamethasone in rats with trigeminal neuropathic pain. The systemic administration of dexamethasone significantly attenuated mechanical allodynia produced by mal-positioned dental implants (data not shown). Dexamethasone also attenuated the increased p-p38 MAPK in the medullary dorsal horn on POD 7, compared to the vehicle-treated group, when administered intraperitoneally once daily for three consecutive days beginning on POD 1 (Figure 6A). Moreover, dexamethasone treatment significantly reduced the expression of p-NF-κB in the medullary dorsal horn on POD 7 compared with the vehicle-treated group (Figure 6B). Western blotting analysis further confirmed the attenuation of p38 MAPK and NF-κB phosphorylation following treatment with dexamethasone (P < 0.05).

## Discussion

The results of our present study demonstrate that mal-positioned dental implants significantly increase the early phosphorylation of p38 MAPK in microglia and late phosphorylation of NF-κB in astrocyte. On POD 3, the intracisternal administration of SB203580 significantly inhibited both mechanical allodynia and the expression of p-p38 MAPK. On POD 21, the intracisternal administration of SN50 significantly inhibited both mechanical allodynia and the expression of p-NF-κB. Moreover, treatment with dexamethasone, which produces potent anti-nociceptive effects, significantly attenuated the expression of p-p38 MAPK and p-NF-κB in rats with inferior nerve injury produced by mal-positioned dental implants. These results suggest that the activation of p38 MAPK and NF-κB play a critical role in the development and maintenance of trigeminal neuropathic pain.

Previous studies have reported that chronic constriction injury of the infraorbital nerve or inferior alveolar nerve damage produces abnormal nociceptive behavior in the trigeminal region [11-15]. These nociceptive behaviors, including allodynia, are similar to the symptoms of patients with neuropathic pain caused by nerve injury. Dental implantation, one of the most common prosthetic treatments, often results in injuries to the nerve including the inferior alveolar or mental nerve. Han et al. have previously described a novel animal model for trigeminal neuropathic pain, which was produced by mal-position dental implants [10]. In our present study, we used this animal model and found that it produced severe long-lasting nociceptive behavior in the ipsilateral and contralateral sides compare to the naïve or sham groups. Accordingly, these results suggest that this is an appropriate animal model for the clinical study of trigeminal neuropathic pain. However, the underlying cellular mechanisms involved in the development and maintenance of trigeminal neuropathic pain remain unclear.

P38 MAPK, one of the families of intracellular signaling molecules that transduce extracellular responses, plays an important role in the development and maintenance of nerve injury-induced pain hypersensitivity [16]. Emerging evidence now indicates that activated p38 MAPK is involved in the development of neuropathic pain after nerve injury. The activation of p38 MAPK has been previously observed in the spinal cord following spinal nerve injury [17-19], spared nerve injury [20], and spinal cord injury [21]. Moreover, the intrathecal administration of a p38 MAPK inhibitor prevents the generation of neuropathic pain in rats with nerve injury [17,18,20,22]. In our present study, the intracisternal administration of SB203580 on POD 3 significantly inhibited mechanical allodynia induced by mal-positioned dental implants. On POD 21 however, SB203580 did not affect mechanical allodynia. This early role of p38 MAPK is consistent with the findings of a previous study showing that the p38 MAPK levels rapidly increase and reached a peak at three days after nerve injury [17]. The results of our present study also show that an inferior alveolar nerve injury markedly induces an increase in p-p38 MAPK expression on POD 3 and 7. Moreover, p38 MAPK immunoreactive cells were found to co-localize with OX42 but not with NeuN or GFAP. These results, taken together with our behavioral analysis, suggest that a blockade of early p38 MAPK expression in microglia may be a potential therapeutic strategy for the treatment of trigeminal neuropathic pain.

Similarly to p38 MAPK, NF-κB is also involved in environmental stress-responsive pathways, including those that are activated by inflammatory insults [23]. Recent studies also indicate that NF-κB is involved in the pathogenesis of neuropathic and inflammatory pain [24-26]. NF-κB is activated in dorsal root ganglia following partial sciatic nerve injury [27] and in the dorsal horn following spinal nerve ligation [7]. The results of our present study demonstrate that the expression of p-NF-κB is significantly increased following nerve injury on POD 7 and 21. The intracisternal injection of SN50, an NF-κB inhibitor, was found to attenuate the expression of p-NF-κB as well as mechanical allodynia on POD 21, but not on POD 3. Moreover the p-NF-κB immunoreactive cells were found to co-localize with GFAP but not with NeuN or OX42. These results suggest that the activation of NF-κB in astrocyte, produced by mal-positioned dental implants, play an important role in the maintenance of trigeminal neuropathic pain. This suggested role of NF-κB in astrocyte in the late stages of neuropathic pain is consistent with the results of previous studies. NF-κB has been shown in an earlier report to mainly co-localize with GFAP [28,29] and the activation of NF-κB in nerve injury models has previously been demonstrated to occur in the later stages of neuropathic pain [7,30]. These results, taken together with our current behavioral analysis, suggest that a blockade of NF-κB in astrocyte may be a potential future therapy for already established trigeminal neuropathic pain.

Spinal glial cells, mainly microglia and astrocyte, play an important role in innate immunity and after peripheral nerve injury are converted to an activated state through a series of cellular and molecular changes [2,3]. Recent studies have also indicated that activated microglia and astrocyte play important roles in the development and maintenance of neuropathic pain caused by nerve injury [7,13,17,31]. Following nerve injury, activated microglia and astrocyte release a variety of pro-inflammatory cytokines, such as IL-1β, IL-6, or TNF-α, which may augment the nociceptive signals in the spinal cord [4]. Moreover, treatment with specific inhibitors of microglia or astrocyte significantly attenuates nociceptive responses following nerve injury [3]. In addition, microglial activation following nerve injury increases in the spinal dorsal horn within several hours [32] whereas astrocyte activation seems to initiate several days after nerve injury [7,31]. However, there is no direct behavioral evidence for the differential regulation of glial cells in rats with trigeminal neuropathic pain. Our current analyses demonstrate that an early blockade of p38MAPK in microglia and a late blockade of NF-κB in astrocyte significantly attenuate mechanical allodynia following inferior alveolar nerve injury. These results suggest that nerve injuries produce a differential activation of spinal glial cells which may contribute to the development or maintenance of trigeminal neuropathic pain.

Previous reports have demonstrated that glucocorticoids attenuate nociceptive behavior in neuropathic pain animal models [33,34] and inhibit the development and maintenance of neuropathic pain [35]. Recently, Han et al. reported that early treatment with dexamethasone produces a prolonged inhibition of mechanical allodynia in a rat model of trigeminal neuropathic pain produced by mal-positioned dental implants [10]. Daily treatment with dexamethasone significantly reduced the expression of p-p38 MAPK and p-NF-κB on POD 7 in our present experiments. Taken together, these results indicate that dexamethasone produces anti-allodynic effects through the inhibition of p38 MAPK and NF-κB activation.

## Conclusions

Our results indicate that the nerve injury produced by mal-positioned dental implants in rats significantly augments the early activation of p38 MAPK in the microglia and the late activation of NF-κB in astrocyte. Early treatment with SB203580, a p38 MAPK inhibitor, and late treatment with SN50, an NF-κB inhibitor, attenuates nociceptive behavior. The antinociceptive effects of dexamethasone are mediated by a blockade of both p38 MAPK and NF-κB activation. These data suggest that the early blockade of p38 MAPK or late inhibition of NF-κB is potential therapeutic approaches to the treatment of trigeminal neuropathic pain produced by dental implants.

## Methods

### Animals and Surgery

Male Sprague-Dawley rats (220-250 g) were used in this study. The animals were anesthetized with 5% isoflurane in oxygen and maintained with 2% during surgery. Under this anesthesia, the left lower second molar was extracted and replaced with a mini dental implant (diameter, 1 mm; length, 4 mm; donated by Megagen, Gyengsan, Korea) to intentionally injure the inferior alveolar nerve as described previously [10]. A sham group was operated on without performing the dental implant placement. All procedures involving the use of animals were approved by the Institutional Care and Use Committee of the School of Dentistry, Kyungpook National University and were carried out in accordance with the ethical guidelines for the investigation of experimental pain in conscious animals of the International Association for the Study of Pain. The animals were maintained in a temperature-controlled room (23 ± 1°C) with a 12/12 hour light-dark cycle. Food and water were freely available. Only data obtained from rats with an inferior alveolar nerve injury caused by mal-positioned dental implants were utilized in the final analyses. Behavioral responses were measured in a blind fashion.

### Evaluation of mechanical allodynia

For behavioral observations, each rat was placed in a customized observation cage in a darkened and noise-free room and the animals were acclimated for at least 30 min. An evaluation of withdrawal behavioral responses was performed after the application of 10 successive trials of constant air-puff pressure (4 sec duration, 10 sec intervals) on freely moving rats as described previously [10,36,37]. The responses observed included escape from air-puff stimulation, aggressive behavior, and biting. The intensity and intervals of the air-puff pressure was controlled with a pneumatic pump module (BH2 system; Harvard Apparatus MA). The air-puffs were applied through a 26-gauge metal tube (length, 10 cm) located 1 cm from the skin at a 90° angle. The air-puff thresholds were determined as the air-puff pressure at which each rat responded in 50% of the trials. The cut-off pressure for the air-puffs was 40 psi. Naïve animals did not respond to a pressure of less than 40 psi.

### Immunohistochemical staining

The rats (n = 3 per group) were transcardially perfused with 0.9% saline, followed by 4% paraformaldehyde in phosphate buffer (0.1 M PB, pH 7.4) on POD 3, 7, and 21. The caudal medullae was dissected out, postfixed in the same fixative at 4°C overnight, and then replaced with 30% sucrose in 0.1 M PB overnight. These tissue samples were then frozen, cut in transverse sections at a 30 μm thickness, and blocked with 5% goat serum in PBS containing 0.2% Triton X-100 for 1 h at room temperature. Subsequently, the specimens were incubated at 4°C overnight with rabbit polyclonal anti-p-p38 MAPK (1:400; Cell Signaling Technology, Danvers, MA) and p-NF-κB (1:400, Cell Signaling Technology, Danvers, MA) antibodies. The sections were subsequently incubated with Alexa 555-conjugated rabbit IgG antibody (1:200, Invitrogen, Carlsbad, CA) for 2 h at room temperature. For double immunofluorescence staining, the sections were incubated with a mixture of p-p38 MAPK or p-NF-κB antibodies with the NeuN, neuronal marker (1:600; Millipore, Temecula, CA), the OX42, microglial marker (1:100, Millipore), or GFAP, astrocytic marker (1:1000; Cell Signaling Technology) overnight at 4°C. This was followed by incubation with a mixture of Alexa 555-conjugated rabbit IgG and Alexa 488-conjugated mouse IgG (1:200; Invitrogen). The stained sections were observed with a fluorescence microscope (BX 41 and U-RFL-T; Olympus, Tokyo, Japan) and a confocal laser scanning microscope (LSM 510; Carl Zeiss, Jena, Germany).

### Western blotting

Animals (n = 5 per group) were sacrificed on POD 3, 7, and 21 by decapitation. The dorsal part of the caudal medulla was then rapidly removed from each rat and frozen in liquid nitrogen. Subsequently, these tissue samples was sonicated with a Biorupture device (Cosmo Bio., Tokyo, Japan) in a lysis buffer containing protease and a phosphatase inhibitor cocktail (Thermo Scientific, Rockford, IL). Protein concentrations in the samples were measured using a fluorometer (Invitrogen). Total proteins (30 μg) were separated in a 4-12% gradient NuPAGE Novex Bis-Tris gel (Invitrogen) and transferred onto PVDF membrane by using an iBlot Dry blotting system (Invitrogen). The membranes were blocked with 5% non-fat milk in TBS containing 0.1% Tween 20 for 1 h at room temperature and then incubated with p-p38 MAPK (1:1000, Cell Signaling Technology) or p-NF-κB (1:500, Cell Signaling Technology) at 4°C overnight. Glyceraldehyde-3-phosphate dehydrogenase (GAPDH) antibody (Santa Cruz Biotechnology, Santa Cruz, CA) was used as a loading control for the p-p38 MAPK and p-NF-κB signals. The blots were then incubated with goat anti-rabbit horseradish peroxidase or goat anti-mouse horseradish peroxidase for 1 h at room temperature. Membranes were developed using the Super-signal West Femto substrate (Pierce, Rockford, IL), and exposed to X-ray film. The ImageJ analysis system (NIH, Bethesda, MD) was used for the quantification of specific bands.

### Drugs administration

To perform intracisternal injections, a polyethylene tube (PE10) was implanted in anesthetized rats three days before conducting the drug tests, as described previously [38-40]. A limited-skin incision was made and part of the atlanto-occipital membrane was exposed by deflecting a part of the muscle from the occipital bone. Using a 27-gauge needle, a tiny opening was made in the dura and the tip of the cannula was inserted through the opening and secured in place with glue. The polyethylene tube was subcutaneously led to the top of the skull and secured in place by a stainless steel screw and dental acrylic resin. Each tube was closed off with a steel wire to prevent leakage of cerebrospinal fluid. We intracisternally administered SB203580 (1 or 10 μg/10 μl), a p38 MAPK inhibitor, or SN50 (0.2 or 2 ng/10 μl), a NF-κB inhibitor, on POD 3 and 21, respectively. After these injections, we evaluated the effects of SB203580 or SN50 on mechanical allodynia in rats with a mal-positioned dental implant. To investigate the antinociceptive mechanisms of dexamethasone, we examined the expression of p-p38 MAPK or p-NF-κB after dexamethasone (25 mg/kg) or vehicle (saline) was intraperitoneally administered daily for three days beginning on POD 1. SB203580 and SN50 were purchased from Calbiochem and dexamethasone was purchased from Sigma. SB203580 was diluted in 70% DMSO. SN50 and dexamethasone were diluted in sterile saline.

### Data analysis

Differences between groups were compared using analysis of repeated measures ANOVA followed by LSD post hoc analysis. Comparisons between two means were performed with a Student's t-test. In all statistical comparisons, P < 0.05 was used as the criterion for statistical significance. All data are presented as the mean ± SEM.

## Abbreviations

p-p38 MAPK: phospho-p38 mitogen-activated protein kinase; p-NF-κB: phospho-nuclear factor- kappa B; IL-1β: interleukin-1 beta; IL6: interleukin-6; TNF-α: tumor necrosis factor-alpha; POD: postoperative day; NeuN: neuronal nuclei; GFAP: glial fibrillary acidic protein; PB: phosphate buffer; GAPDH: Glyceraldehyde-3-phosphate dehydrogenase.

## Competing interests

The author declares that they have no competing interests.

## Authors' contributions

MKL and SRH carried out the experiment and drafted the manuscript. MKP, MJK, SKL and JSP participated in the design of the study. YCB help conceive of the study, and participated in its design. DKA coordinated and supervised the experiments, analyzed the data and wrote the manuscript. All authors read and approved the final manuscript.
